# Genetic ablation of myeloid integrin α9 attenuates early atherosclerosis

**DOI:** 10.1093/jleuko/qiae161

**Published:** 2024-07-22

**Authors:** Tarun Barbhuyan, Rakesh B Patel, Ivan Budnik, Anil K Chauhan

**Affiliations:** Division of Hematology/Oncology, Department of Internal Medicine, University of Iowa, 3160 Medical Labs, Iowa City, IA 52242, United States; Division of Hematology/Oncology, Department of Internal Medicine, University of Iowa, 3160 Medical Labs, Iowa City, IA 52242, United States; Division of Hematology/Oncology, Department of Internal Medicine, University of Iowa, 3160 Medical Labs, Iowa City, IA 52242, United States; Division of Hematology/Oncology, Department of Internal Medicine, University of Iowa, 3160 Medical Labs, Iowa City, IA 52242, United States

**Keywords:** atherosclerosis, integrin α9β1, neutrophil extracellular traps, neutrophils

## Abstract

Integrin α9β1 is known to stabilize leukocyte adhesion to the activated endothelium. We determined the role of myeloid cell α9β1 in early atherosclerosis in two models: *α9^Mye-KO^Apoe^−/−^* or the *Ldlr^−/−^* mice transplanted with bone marrow (BM) from *α9^Mye-KO^* mice fed a high-fat “Western” diet for 4 wk. *α9^Mye-KO^Apoe^−/−^* mice exhibited reduced early lesions in the aortae and aortic sinuses (*P* < 0.05 vs *α9^WT^ Apoe^−/−^* mice). Similar results were obtained in *α9^Mye-KO^ BM→Ldlr^−/−^* mice (*P* < 0.05 vs *α9^WT^ BM→Ldlr^−/−^* mice). Reduced early atherosclerosis in *α9^Mye-KO^Apoe^−/−^* mice was associated with decreased neutrophil and neutrophil extracellular traps (NETs) content in the aortic lesions (*P* < 0.05 vs *α9^WT^Apoe^−/−^*). Vascular cell adhesion molecule-1-stimulated neutrophils from *α9^Mye-KO^* mice exhibited reduced adhesion, transmigration, and NETs formation (NETosis) (*P* < 0.05 vs *α9^WT^* neutrophils). Reduced NETosis was associated with decreased extracellular signal-regulated kinase phosphorylation, peptidyl arginine deiminase 4, and citrullinated histone H3 expression. In summary, genetic ablation of myeloid cell-specific α9 reduces early atherosclerosis, most likely by reducing neutrophil adhesion, transmigration, and NETosis.

## Teaser

Utilizing atherosclerosis-prone mice, we demonstrated that myeloid integrin α9β1 contributes to early atherosclerosis, most likely by potentiating neutrophil recruitment and neutrophil extracellular traps formation.

## 1. Introduction

Atherosclerosis is one of the primary causes of coronary artery disease. The role of monocytes/macrophages in all stages of atherosclerosis is well characterized, while the role of neutrophils is understudied due to their sparse presence in human atherosclerotic lesions. In contrast, murine studies suggest the presence of neutrophils in the early atherosclerosis, particularly in the plaque shoulder region in the apolipoprotein E-deficient (*Apoe^−/−^*) mice fed a high-fat “Western” diet for 4 wk.^1–3^ In proof-of-concept studies, neutrophil depletion reduced early atherosclerosis in *Apoe^−/−^* mice.^1^ Neutrophil extracellular traps (NETs) are known to contribute to early atherosclerosis by potentiating inflammation.^2,4,5^ Integrin α9β1 present on myeloid cells mediates leukocyte adhesion to the activated endothelium in synergy with β2 integrins.^6^ Integrin α9β1 closely resembles α4β1; however, murine studies using α9- or α4-deficient mice demonstrated different phenotypes, suggesting unique functions of the integrins in vivo.^7,8^ We have previously demonstrated that α9β1 is upregulated more on neutrophils than monocytes and contributes to thromboinflammation.^9–11^ A previous study showed that α9 deletion in myeloid cells did not reduce advanced atherosclerotic lesions in *Apoe^−/−^* mice.^12^ However, it remains unclear whether myeloid-specific α9β1 contributes to early atherosclerosis. In the present study, using myeloid-specific integrin subunit α9-deficient mice on the *Apoe^−/−^* or *Ldlr^−/−^* background, we delineated the role of integrin α9β1 present on the surface of myeloid cells in developing early atherosclerosis.

## 2. Materials and methods

### 2.1 Mice

Six-wk-old female *Apoe^−/−^* and *Ldlr^−/−^* mice were used in the present study. *α9^fl/fl^* mice were crossed with *LysMcre^+/−^* to generate myeloid cell-specific α9-deficient mice (*α9^fl/fl^LysMcre^+/−^*).^10^*α9^fl/fl^Apoe^−/−^* mice were crossed with *LysMcre^+/−^Apoe^−/−^* mice to generate *α9^fl/fl^LysMcre^+/−^Apoe^−/−^*.^10^ Experimental mice and littermates were obtained by crossing *α9^fl/fl^LysMcre^+/−^Apoe^−/−^* to *α9^fl/fl^LysMcre^−/−^Apoe^−/−^*. Mice were genotyped by polymerase chain reaction (PCR) as described previously.^9^

### 2.2 Animal diet feeding and preparation of tissues

All mice were fed a high-fat “Western” diet (20% milk fat and 0.2% cholesterol, Teklad) for 4 wk beginning at 10 wk of age (4 wk following bone marrow [BM] transplantation) for *Ldlr^−/−^* mice and 6 wk of age for mice on the *Apoe^−/−^* background. Blood samples were collected in heparinized tubes following overnight fasting by retroorbital plexus puncture. Before being sacrificed, the anesthetized mice were perfused via the left ventricle with 10 mL of phosphate-buffered saline (PBS) containing heparin followed by 10 mL of 4% paraformaldehyde (PFA). After perfusion, the aorta was isolated and dehydrated for 5 min in 60% isopropanol for *en-face* lesion analysis. Heart tissues with aortic roots were dissected and fixed overnight in 4% PFA before embedding in paraffin.

### 2.3 Immunofluorescence staining of the aortic root

Aortic root sections (frozen) were fixed with 4% PFA for 10 min, followed by permeabilization in 0.2% Triton X-100 for 8 min and blocked in PBS containing 3% bovine serum albumin (BSA) for 1 h at room temperature. Sections were then stained with antibodies specific for neutrophils (Ly6G) (Abcam, catalog #ab210204, diluted 1:100) and citrullinated histone H3 (H3Cit) (Abcam, catalog #ab61251, diluted 1:100). The sections were washed and labeled with the Alexa Fluor 568-conjugated (Thermo Fisher Scientific, catalog #A-11004, diluted 1:500) and fluorescein isothiocyanate-conjugated secondary antibody (Southern Biotech, catalog #4052-02, diluted 1:500) for 1 h at room temperature. Sections were counterstained with Hoechst (2 μg/mL) before mounting. The Ly6G- and H3Cit-positive areas were quantified using ImageJ (NIH ImageJ, USA) and expressed as % of the total lesion area. The NETs area was determined as the area showing double positivity for Ly6G and H3Cit. Few sections were stained with antibodies specific for macrophages (Mac-3) (BD Pharmingen, catalog #553322, diluted 1:100) and vascular cell adhesion molecule-1 (VCAM-1) (Santa Cruz, catalog #sc-13160, diluted 1:100) to examine the extent of VCAM-1/Mac-3 co-localization within the lesion area.

### 2.4 Neutrophil isolation

BM-derived neutrophils were isolated from female *α9^fl/fl^LysMcre^+/−^* or *α9^fl/fl^LysMcre^−/−^* mice as described.^13^ Femurs and tibias were removed aseptically, BM cavities were flushed, and cells were washed with high-glucose Dulbecco's modified Eagle's medium (DMEM) containing 25 mM glucose, 4 mM glutamine, 1 mM pyruvate, 10% fetal calf serum, 1% penicillin, and 1% streptomycin. After red blood cell lysis, neutrophils were isolated by density gradient centrifugation using Histopaque 1119 (density, 1.119 g/mL; Sigma, catalog #11191) and Histopaque 1077 (density, 1.077 g/mL; Sigma, catalog #10771).

### 2.5 Adhesion

Static adhesion assays were performed in 24-well plates coated with 20 µg/mL soluble VCAM-1 (sVCAM-1). The wells were washed with PBS and blocked with 1% BSA in Roswell Park Memorial Institute (RPMI)-1640 at 37 °C for 1 h. The control wells were filled with 1% BSA in RPMI-1640. Mouse neutrophils (6 × 10^5^ cells in RPMI-1640) were seeded into each well of a 24-well plate and allowed to adhere for 1 h (37 °C, 5% CO_2_). Following incubation, the cells that had not adhered to the wells were discarded, and the wells were washed twice with PBS, fixed in 4% PFA, and stained with 0.5% crystal violet. Images of the cells were captured, and cell numbers were counted under a microscope (Olympus Corporation) at a magnification of ×100.

### 2.6 Transwell cell migration assay

Stimulated migration and chemotaxis were measured using 6.5-mm Transwell with 5.0-µm pore polycarbonate membrane insert (Corning, catalog #3421). Neutrophils (6 × 10^5^) were incubated with sVCAM-1 (20 µg/mL) for 1 h at 37 °C, then placed in the upper chamber, and then allowed to migrate at 37 °C to the lower chamber containing *N*-formyl-L-methionyl-L-leucyl-L-phenylalanine (fMLP; 10 µM; Sigma, catalog #47729) overnight. Control experiments were conducted without sVCAM-1 treatment in the cell culture medium or medium containing fMLP. The neutrophils in the upper chamber were removed, and the neutrophils that had migrated into the lower chamber were collected. To assess their viability, neutrophils were stained with trypan blue and counted using an automatic cell counter.

### 2.7 NETs formation

The BM-derived neutrophils were seeded at a density of 2 to 3 × 10^5^ cells/mL on 12-mm coverslips coated with vehicle or sVCAM-1 (20 µg/mL) for 4 h at 37 °C. Neutrophils were pretreated with 1 μM BIO5192 (R&D Systems, catalog #5051) while seeding. The cells were then fixed with 4% PFA for 15 min at room temperature, and then, SYTOX Green (Thermo Fisher Scientific, catalog #S7020) and Hoechst 33342 were added. NETs stained by SYTOX Green were visualized and identified as extracellular DNA with fibrous structures by fluorescence microscopy. To calculate the percentage of NETs formation (NETosis), the number of SYTOX Green-positive cells was divided by the total number of neutrophils.

### 2.8 Statistical analysis

Statistical analysis was performed using GraphPad Prism (version 10.0.3 for Windows, GraphPad Software, Boston, MA, USA). The data were presented as mean ± standard error of the mean (SEM). The Shapiro–Wilk test was used to assess normality. The statistical significance of the difference between the two means was assessed using the unpaired two-tailed *t*-test or a two-way ANOVA as appropriate. *P* < 0.05 was considered statistically significant.

## 3. Results

### 3.1 Myeloid-specific α9-deficient mice are less susceptible to early atherosclerosis

**Fig. 1.**
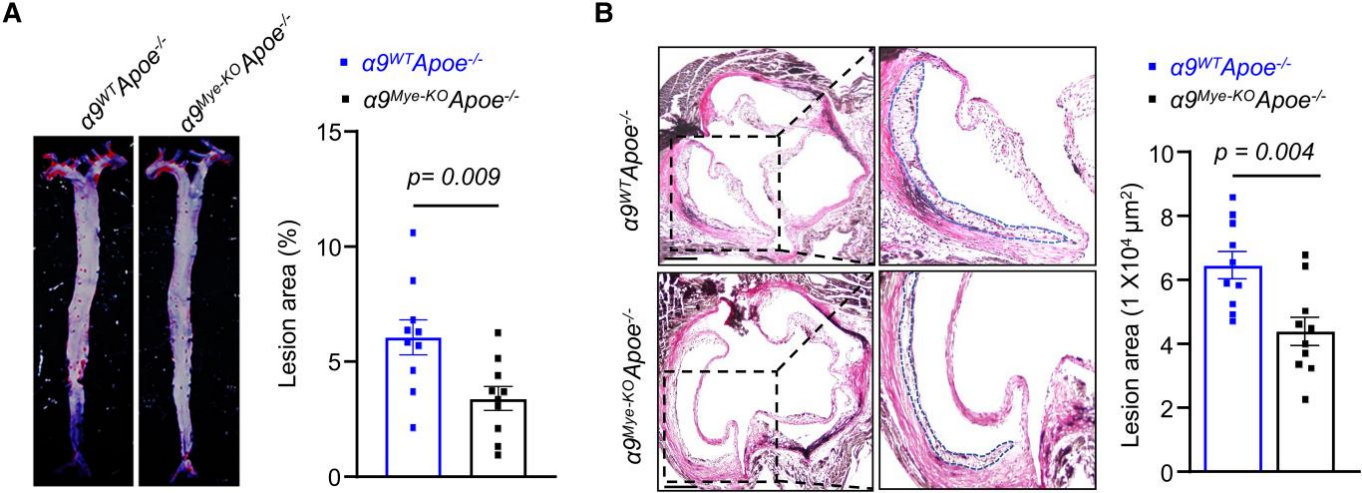
Genetic ablation of α9 in myeloid cells reduces early atherosclerosis in the *Apoe^−/−^* mice. All the mice were females and fed a high-fat diet for 4 wk. A) Representative photomicrographs and quantification of Oil Red O-stained *en-face* lesion areas in the whole aortae (*n* = 10 mice/group). B) Representative photomicrographs and quantification of Verhoeff–van Gieson staining in aortic sinuses in the *Apoe^−/−^* mice (*n* = 10/group). Higher-magnification images showing atherosclerotic lesions from the boxed region. Scale bar: 500μm. The data are presented as mean ± SEM. Statistical analysis: unpaired two-tailed *t*-test.

To determine the role of myeloid-specific α9β1 in early atherosclerosis, we crossed *α9^fl/fl^LysMcre^+/−^* mice with *Apoe^−/−^* mice. The resulting *α9^fl/fl^LysMcre^+/−^Apoe^−/−^* mice and littermate control *α9^fl/fl^LysMcre^−/−^Apoe^−/−^* mice (from now on referred to as *α9^Mye-KO^Apoe^−/−^* and *α9^WT^Apoe^−/−^* mice, respectively) were fed a high-fat “Western” diet for 4 wk, beginning at 6 wk of age, and evaluated for atherosclerotic burden. We found that the mean lesion area in the aortae and aortic sinuses was significantly decreased in *α9^Mye-KO^Apoe^−/−^* mice (*P* < 0.05 vs *α9^WT^Apoe^−/−^* mice; Fig. 1). Notably, the plasma levels of TNF-α or IL-1β were comparable between the two mouse groups (Supplementary Fig. 1).

**Fig. 2.**
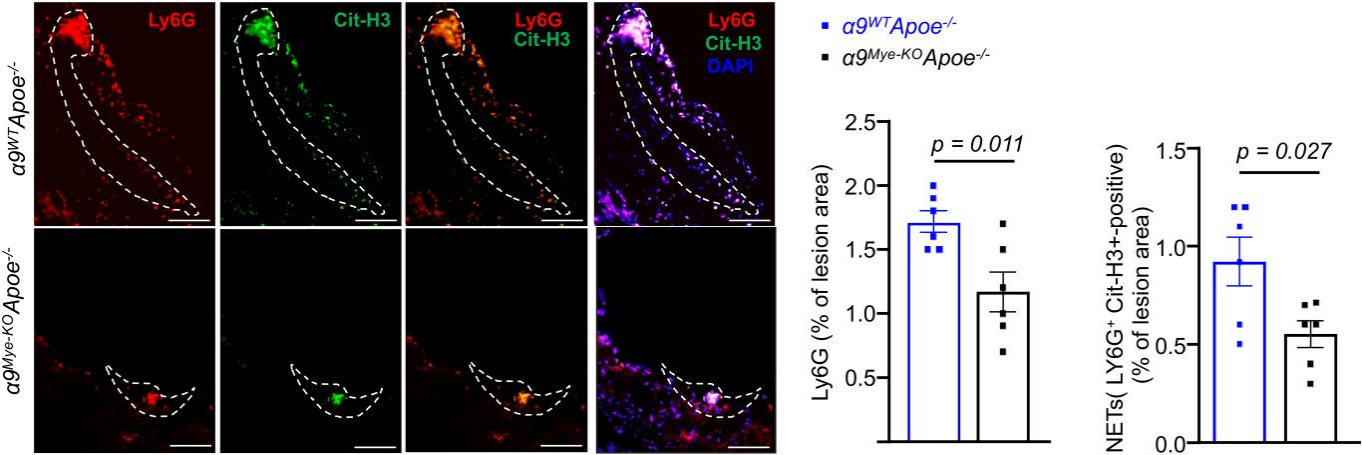
*α9^Mye-KO^Apoe^−/−^* mice exhibits decreased content of neutrophils and NETs in the aortic root. All the mice were females and fed a high-fat diet for 4 wk. Representative photomicrographs and quantification of neutrophils (Ly6G-positive area) and NETs (H3Cit-positive area) within plaques in female *Apoe^−/−^* mice (*n* = 6/group). Scale bar: 100 μm. The lesions are demarcated by white dotted lines. The data are presented as mean ± SEM. Statistical analysis: unpaired two-tailed *t*-test.

Previous studies have demonstrated that neutrophils contribute to early atherosclerosis by releasing NETs.^2,4,5^ Therefore, we examined whether the decrease in atherosclerotic lesion size in *α9^Mye-KO^Apoe^−/−^* mice was associated with decreased content of neutrophils and NETs in the aortic root. Immunohistochemical staining revealed that *α9^Mye-KO^Apoe^−/−^* mice exhibited significantly reduced neutrophil and NETs content (the Ly6G- and H3Cit-positive areas, respectively) within the lesions, particularly in the shoulder region (*P* < 0.05 vs *α9^WT^Apoe^−/−^* mice; Fig. 2).

To determine whether the regulatory role of myeloid-specific α9 in early atherosclerosis was model-dependent (*Apoe^−/−^* background) or not, we generated an additional model in which irradiated *Ldlr^−/−^* mice were reconstituted with the BM cells from either *α9^Mye-KO^ (α9^fl/fl^LysMcre^+/−^)* or *α9^WT^ (α9^fl/fl^LysMcre^−/−^)* mice. Four weeks after the transplantation, successful engraftment of the BM was confirmed by genomic PCR of peripheral myeloid cells (not shown). The mice were then switched from a standard diet to the high-fat “Western” diet for 4 wk. Total cholesterol, triglyceride levels, and complete blood count were comparable between the groups (Supplementary Tables 1–3). The mean lesion area in the aortic sinuses was significantly decreased in *α9^Mye-KO^ BM→Ldlr^−/−^* mice (*P* < 0.05 vs *α9^WT^ BM→Ldlr^−/−^* mice; Supplementary Fig. 2). Similar results were obtained in male mice (Supplementary Fig. 2), ruling out the effect of sex on the role of myeloid α9 in early atherosclerosis.

### 3.2 Integrin α9β1–VCAM-1 interaction contributes to neutrophil adhesion, transmigration, and NETosis

**Fig. 3.**
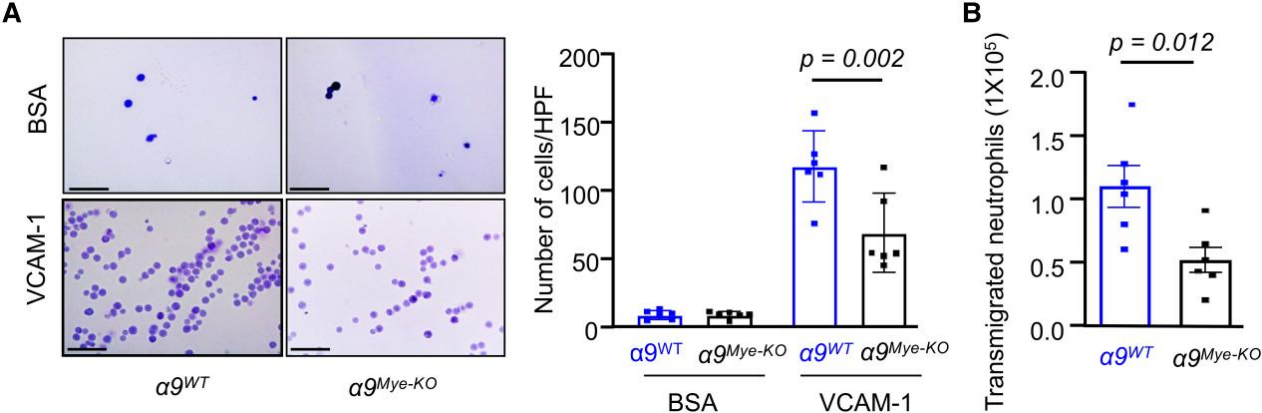
α9-deficient neutrophils exhibit reduced adhesion and transmigration. A) BM-derived neutrophils were stimulated with sVCAM-1 (20 µg/mL) for 1 h at 37 °C and subjected to an adhesion assay (*n* = 6/group).Scale bar: 100 μm. B) BM-derived neutrophils were stimulated with sVCAM-1 (20 µg/mL) for 1 h at 37 °C and subjected to a transmigration assay (*n* = 6/group). The data are from female mice and presented as mean ± SEM. Statistical analysis: two-way ANOVA (A) and unpaired two-tailed *t*-test (B). HPF, high-power field.

Integrin α9β1 is known to promote neutrophil adhesion and transmigration across the endothelial lining, which at least in part is due to its interaction with multiple ligands, including VCAM-1, tenascin C, and a few more ligands.^14^ Evidence from murine studies suggested that VCAM-1 contributes to early atherosclerosis.^15^ Therefore, we examined whether VCAM-1 could be an α9β1 ligand that may potentiate neutrophil adhesion, transmigration, NETosis, and thereby early atherosclerosis. To explore this possibility, we stimulated *α9^Mye-KO^* and *α9^WT^* BM-derived neutrophils with recombinant sVCAM-1 ex vivo. The results showed that sVCAM-1-stimulated *α9^Mye-KO^* neutrophils exhibited reduced adhesion and transmigration (*P* < 0.05 vs sVCAM-1-stimulated *α9^WT^* neutrophils; Fig. 3). To determine whether this effect was specific to sVCAM-1, we also assessed transmigration in response to fMLP, a potent neutrophil chemoattractant binding to formyl peptide receptors. Interestingly, fMLP-induced transmigration was also reduced in *α9^Mye-KO^* neutrophils (*P* < 0.01 vs *α9^WT^* neutrophils; Supplementary Fig. 3), suggesting that the impairment in neutrophil transmigration in the absence of α9β1 is a more general phenomenon.

**Fig. 4.**
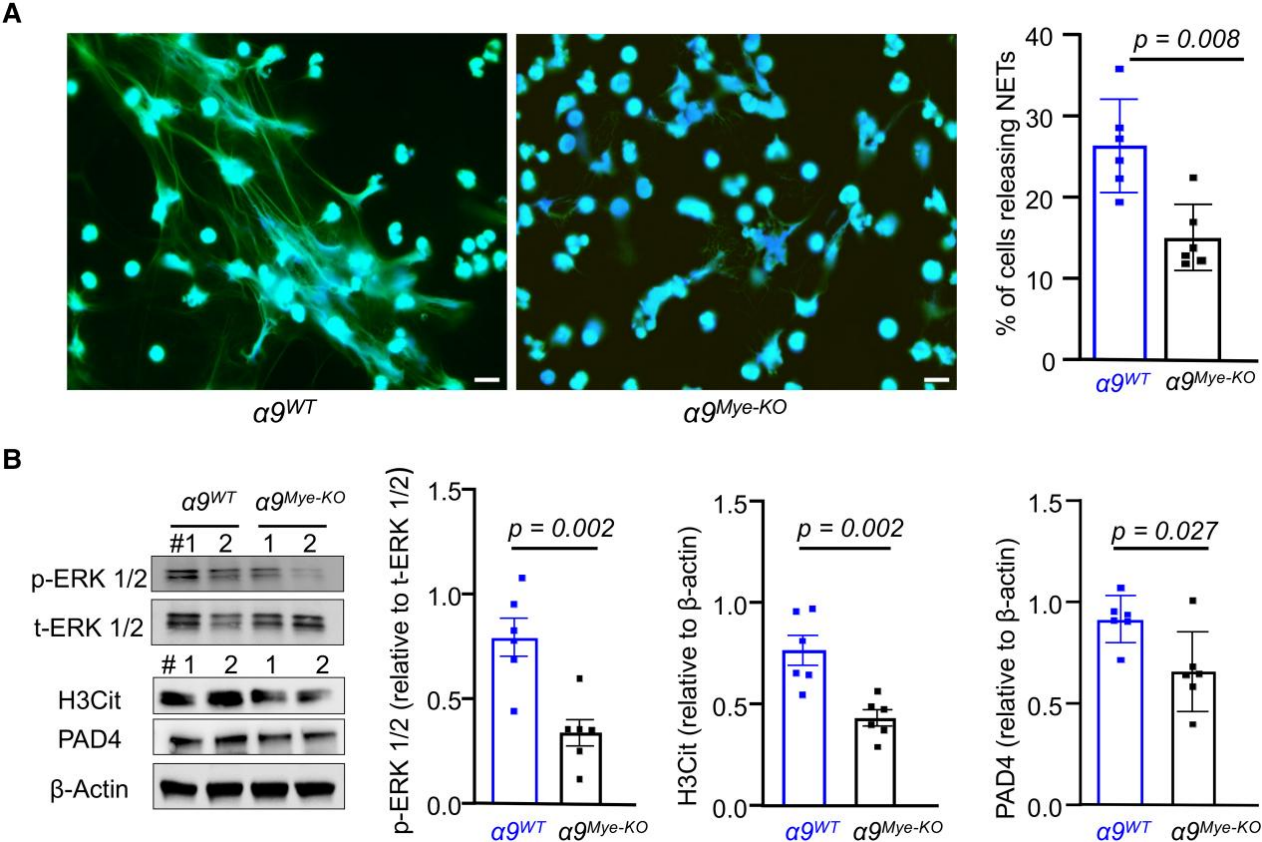
α9-deficient neutrophils exhibit reduced NETosis. A) Neutrophils were stimulated with sVCAM-1 (20 µg/mL) for 4 h at 37 °C and stained with SYTOX Green nucleic acid strain (NETs-positive cells stained as green) and counterstained with Hoechst (blue). Scale bar: 10 μm. The percentage of neutrophils releasing NETs was calculated as the amount of NETs/number of neutrophils (Hoechst-positive) × 100 (*n* = 6/group). B) Neutrophils were stimulated with sVCAM-1 (20 µg/mL) for 15 min (for ERK1/2) or 4 h (for H3Cit and PAD4). Representative immunoblots and densitometric analysis of p-ERK1/2, H3Cit, and PAD4 in neutrophil lysates. β-Actin was used as a loading control for H3Cit and PAD4 (*n* = 6/group). The data are from female mice and presented as mean ± SEM. Statistical analysis: unpaired two-tailed *t*-test.

Next, we determined whether α9β1–VCAM-1 interaction potentiates NETosis. Our results showed that NETosis was significantly reduced in the sVCAM-1-stimulated *α9^Mye-KO^* neutrophils (*P* < 0.05 vs sVCAM-1-stimulated *α9^WT^* neutrophils; Fig. 4A). Without stimulation, the percentage of neutrophils releasing NETs was minimal and comparable (Supplementary Fig. 4). The fact that some NETosis still occurred following stimulation of *α9^Mye-KO^* neutrophils with sVCAM-1 implies that other VCAM-1-binding receptors on neutrophils, such as integrin α4β1, may mediate their residual function.^16^ To explore this possibility, we repeated this experiment in the presence of BIO5192, a highly potent and selective inhibitor of integrin α4β1, and found that pretreatment with the inhibitor reduced sVCAM-1-stimulated NETosis in both *α9^WT^* and *α9^Mye-KO^* neutrophils (Supplementary Fig. 5), suggesting that integrin α4β1 also contributes to VCAM-1-mediated NETosis.

To elucidate the mechanisms by which the α9β1–VCAM-1 interaction facilitates NETosis, we investigate the phosphorylation of extracellular signal-regulated kinase (ERK), a molecule implicated in suicidal NETosis and involved in α9β1 signaling,^17,18^ in sVCAM-1-stimulated *α9^Mye-KO^* and *α9^WT^* BM-derived neutrophils. Additionally, we assessed the expression levels of peptidyl arginine deiminase 4 (PAD4) and H3Cit, which serve as general markers of NETosis. Our results showed that sVCAM-1-stimulated *α9^Mye-KO^* neutrophils exhibited significantly reduced ERK phosphorylation and lower PAD4 and H3Cit levels (*P* < 0.05 vs sVCAM-1-stimulated *α9^WT^* neutrophils; Fig. 4B).

**Fig. 5.**
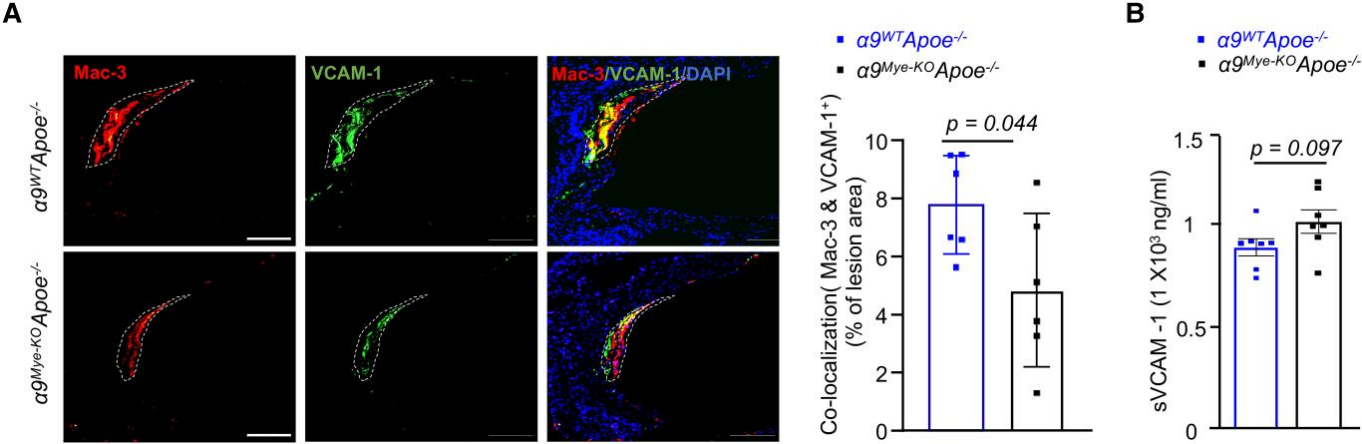
*α9^Mye-KO^Apoe^−/−^* mice exhibit reduced VCAM-1/Mac-3 co-localization in the aortic root. All the mice were females and fed a high-fat diet for 4 wk. A) Representative photomicrographs and quantification of Mac-3-positive area (red) and VCAM-1-positive area (green) within plaques in female *Apoe^−/−^* mice (*n* = 6/group). Nuclei were counterstained with DAPI (blue). Scale bar: 100 μm. The lesions are demarcated by white dotted lines. B) Quantification of sVCAM-1 in the plasma (*n* = 7/group). The data are presented as mean ± SEM. Statistical analysis: unpaired two-tailed *t*-test.

To determine whether sVCAM-1 or insoluble (cell-associated) VCAM-1 mediates α9β1-dependent early atherosclerosis in vivo, we examined the co-localization of VCAM-1 with Mac-3, a marker for mature macrophages, in atherosclerotic plaques from *α9^Mye-KO^Apoe^−/−^* and control *α9^WT^Apoe^−/−^* mice. Immunofluorescence staining revealed that VCAM-1 co-localized with Mac-3 positive macrophages in the lesions of both genotypes, indicating the presence of cell-associated VCAM-1 on plaque macrophages. Notably, the extent of VCAM-1/Mac-3 co-localization was reduced in plaques from *α9^Mye-KO^Apoe^−/−^* mice (*P* < 0.05 vs *α9^WT^Apoe^−/−^* mice; Fig. 5A), suggesting that the absence of α9β1 integrin in myeloid cells leads to decreased accumulation of cell-associated VCAM-1 in atherosclerotic lesions. In contrast to the differential expression of cell-associated VCAM-1, plasma levels of sVCAM-1 were comparable between the two genotypes (Fig. 5B).

## 4. Discussion

In the present study, using myeloid-specific integrin subunit α9-deficient mice, we delineated the role of α9β1 present on the surface of myeloid cells in developing early atherosclerosis. It is important to note that out of the eight β subunits, β1 is the only subunit known to pair with α9, suggesting that α9 deletion in myeloid cells should completely prevent the expression of α9β1 without affecting other integrins pairing with β1 subunit, including α2β1, α4β1, α5β1, and α6β1.^19^ Thus, the α9-deficient genetic model eliminates integrin α9β1 expression, allowing us to examine its unique contribution to atherosclerosis pathophysiology while preserving the function of other integrins in myeloid cells.

Utilizing atherosclerosis-susceptible *Apoe^−/−^* and *Ldlr^−/−^* mice fed a high-fat diet for 4 wk, we demonstrated that genetic ablation of integrin α9β1 in myeloid cells resulted in a significant reduction in early atherosclerotic lesions. However, a previous study^12^ demonstrated that genetic ablation of α9β1 in myeloid cells did not affect the atherosclerotic burden in *Apoe^−/−^* mice fed a high-fat diet for 8 or 16 wk. A possible reason for such incongruity is that atherosclerotic lesions in that study were evaluated at later stages of the disease progression, i.e. at the time points when other pathogenetic factors might have also contributed to plaque formation and overcome the effect of α9β1 ablation. At the same time, our results are in line with those of another study^1^ showing that depletion of neutrophils considerably reduced atherosclerotic lesions (by ∼49%) in *Apoe^−/−^* mice fed a high-fat diet for 4 wk but had no effect in *Apoe^−/−^* mice fed the same diet for 16 wk, suggesting that other cell types, including monocytes and/or smooth muscle cells, drive atherosclerosis progression at later stages. These observations support our hypothesis that integrin α9β1 on myeloid cells, most likely on neutrophils, contributes to the initiation of atherosclerosis.

The reduction in atherosclerotic burden in myeloid cell α9-deficient mice was not accompanied by a significant change in plasma total cholesterol and triglyceride levels, implying that a theoretical alteration in lipid metabolism could not explain the atheroprotective effect. These findings agree with other studies, which showed that the severity of atherosclerosis lesions does not always correlate with the extent of plasma cholesterol and LDL increase.^20–23^ Additionally, no difference in plasma TNF-α or IL-1β levels was found between the *α9^Mye-KO^Apoe^−/−^* and *α9^WT^Apoe^−/−^* mice, suggesting that the observed atheroprotection in the *α9^Mye-KO^Apoe^−/−^* mice was not due to a decrease in the production of proinflammatory cytokines, the known promoters of atherosclerosis.^24,25^

Neutrophils play a crucial role in the development of early atherosclerosis by promoting aseptic inflammation in the arterial wall prone to lesion formation.^1,2^ The reduced neutrophil count in the plaques of *α9^Mye-KO^Apoe^−/−^* mice implies a mechanistic role for neutrophil α9β1 in neutrophil recruitment during the initial stage of atherosclerosis. Among the known α9β1 ligands, VCAM-1 appears to be the most relevant in regulating neutrophil recruitment and function in this context, as it is predominantly expressed on the surface of activated endothelium and other cells in the atherosclerotic lesion, such as macrophages and smooth muscle cells.^15,26,27^ Our ex vivo experiments demonstrated that *α9^Mye-KO^* neutrophils exhibited reduced adhesion and transmigration when stimulated with sVCAM-1. While other α9β1 ligands, including Fn-EDA, may also contribute to early atherosclerosis, the significant reduction in lesions observed in *α9^Mye-KO^Apoe^−/−^* mice can be at least partially attributed to decreased VCAM-1-mediated neutrophil recruitment. Interestingly, the impaired transmigration of *α9^Mye-KO^* neutrophils was not limited to sVCAM-1 stimulation, as fMLP-induced transmigration was also reduced in these cells. These findings indicate that the defect in neutrophil transmigration in the absence of α9β1 is a more general phenomenon, not restricted to sVCAM-1 stimulation. This suggests that α9β1 may regulate neutrophil migration through mechanisms beyond its direct interaction with VCAM-1 or other ligands, possibly by modulating signaling pathways or cytoskeleton reorganization involved in cell motility.

Evidence from experimental murine models suggests that NETosis is one of the mechanisms by which neutrophils contribute to early atherosclerosis.^4,5^ Our ex vivo experiments demonstrated that *α9^Mye-KO^* neutrophils exhibited reduced NETosis when stimulated with sVCAM-1.

The residual NETosis observed in these cells implies the involvement of other VCAM-1-binding receptors on their surface, such as integrin α4β1, in mediating this process.^16^ To explore this possibility, we inhibited integrin α4β1 using BIO5192 and found that NETosis was further reduced, indicating that integrin α4β1 might also contribute to sVCAM-1-stimulated NETosis, particularly in the absence of α9β1. However, the incomplete suppression of NETosis in the presence of BIO5192 suggests that additional VCAM-1-binding neutrophil receptors beyond α9β1 and α4β1 may also be involved in this process. Further research is required to identify these receptors and elucidate their role in NETosis and early atherosclerosis development.

It has been demonstrated that upon recruitment to the growing plaque, neutrophils undergo activation characterized by the generation of reactive oxygen species via the NADPH oxidase activity, a process associated with suicidal rather than vital NETosis (reviewed).^28^ Considering that suicidal NETosis requires the induction of the ERK signaling pathway^17^ and this pathway can be induced via the ligation of integrin α9β1,^18^ we hypothesized that integrin α9β1 can mediate suicidal NETosis in atherosclerosis. Our results showed that sVCAM-1-stimulated *α9^Mye-KO^* neutrophils exhibited reduced ERK phosphorylation as well as lower PAD4 and H3Cit levels, the markers of overall NETosis activity. These findings suggest that the VCAM-1/α9β1/ERK axis mediates suicidal NETosis, thereby identifying a distinct molecular process potentially involved in propagating arterial damage underlying early atherogenesis.

The present study also demonstrated that cell-associated VCAM-1, rather than sVCAM-1, plays a crucial role in mediating α9β1-dependent early atherosclerosis, as evidenced by reduced VCAM-1/Mac-3 co-localization in *α9^Mye-KO^Apoe^−/−^* plaques. This phenomenon is likely a secondary effect of the impaired neutrophil infiltration and NETosis caused by the lack of integrin α9β1 on their surface. The resulting less inflammatory microenvironment could indirectly lead to reduced macrophage infiltration and/or VCAM-1 expression in the lesions. Taken together, these findings suggest that the VCAM-1 expressed on plaque macrophages, and potentially on endothelial cells, rather than sVCAM-1 in the circulation, mediates α9β1-dependent neutrophil recruitment and NETosis in early atherosclerosis.

The strengths of the study include using two different atherosclerosis-prone mouse models (*Apoe^−/−^* and *Ldlr^−/−^* mice) to examine the role of myeloid cell-specific α9β1 in the development of early atherogenesis. A limitation of the study is that the neutrophil-specific *α9^−/−^* mice were not used to examine the specific role of neutrophil α9β1 in early atherosclerosis.

## Conclusion

In conclusion, we propose a mechanistic pathway in which the interaction between integrin α9β1 and VCAM-1 may mediate neutrophil recruitment followed by enhanced NETosis, most likely via the ERK signaling cascade, thereby promoting early atherosclerosis. Thus, precisely targeting the VCAM-1/α9β/ERK axis could represent a novel therapeutic approach for suppressing early atherosclerotic lesions by reducing neutrophil infiltration and subsequent NETosis damage to the arterial wall.

## Supplementary Material

qiae161_Supplementary_Data
