# Supplementary material for: Genetic ablation of myeloid integrin α9 attenuates early atherosclerosis
Source: J Leukoc Biol. 2024 Jul 22;116(5):1208–14. doi: 10.1093/jleuko/qiae161 (PMC11531806; doi:10.1093/jleuko/qiae161)
Supplement: qiae161_Supplementary_Data [file qiae161_supplementary_data.pdf]

Online Supplement

Genetic ablation of myeloid integrin α9 attenuates early atherosclerosis

Tarun Barbhuyan, Rakesh B. Patel, Ivan Budnik, and Anil K. Chauhan
Department of Internal Medicine, Division of Hematology/Oncology, University of Iowa, Iowa City, Iowa, USA.

Running title: **Myeloid integrin α9 promotes early atherosclerosis**

Methods:

Quantification of atherosclerotic lesions

The whole aortae were isolated and stained with Oil Red O, and the *en-face* lesion area was measured using the ImageJ software (NIH ImageJ, USA) as described.[1] Lesion areas in the aortic sinus were quantified using 5-µm thick serial cross-sections cut through the aorta beginning at the origin of the aortic valve leaflets and stained by the Verhoeff–Van Gieson method. The cross-sectional lesion area from each mouse was calculated by taking the mean value of 4 sections (each 80 µm apart, beginning at the aortic valve leaflets and spanning 320 µm) as described previously.[1]

Bone marrow transplantation

Six-week-old *Ldlr*$^{-/-}$ mice were used for bone marrow (BM) transplantation experiments. Recipient mice were irradiated with two doses of 6.5 Gy each separated by a 4-hour interval. The BM cells were extracted from the femurs and tibias of euthanized donor mice under sterile conditions. The BM cells (1×10^7) were suspended in 200 µL sterile PBS and injected into the retroorbital venous plexus of lethally irradiated recipient mice. Female *α9*$^{fl/fl}$*LysMcre*$^{+/-}$or *α9*$^{fl/fl}$*LysMcre*$^{-/-}$ BM cells were transplanted to female *Ldlr*$^{-/-}$ mice. Similarly, male

α9$^{fl/fl}$*LysMcre*$^{+/−}$or *α9*$^{fl/fl}$*LysMcre*$^{−/−}$ BM cells were transplanted to male *Ldlr*$^{−/−}$ mice. Mice were maintained in sterile cages and fed autoclaved food and water *ad libitum*. Successful BM transplantation was confirmed after four weeks by PCR to check for the presence of genomic DNA (of the respective donor mice) in peripheral blood mononuclear cells from the transplanted mice (not shown). Complete blood counts were obtained using the automated veterinary analyzer ADVIA 120 Hematology System to confirm that BM transplantation did not affect the number of BM-derived blood cells.

Western blot

BM-derived neutrophils were stimulated with sVCAM-1 (20 µg/mL) for 15 minutes (for ERK1/2) or 4 h (for H3Cit and PAD4) at 37°C. Neutrophils were lysed and then sonicated in ice-cold radioimmunoprecipitation assay (RIPA) buffer containing proteinase inhibitors. The lysates were centrifuged at 14,000 rpm for 15 minutes at 4°C, and the supernatant was collected. Protein quantification was performed by the Bradford method. An equal amount of total protein was subjected to SDS-PAGE (sodium dodecyl sulfate-polyacrylamide gel electrophoresis) using 12% or 4–20% gels. The proteins were then transferred onto polyvinylidene fluoride (PVDF) membranes by using a Bio-Rad Western blotting system. The membranes were blocked with 5% BSA for 1 h at room temperature and incubated with primary antibodies against phosphorylated ERK1/2 (p-ERK1/2; 1:1000; Cell Signaling Technology, catalog #4370), total ERK1/2 (t-ERK; 1:1000; Cell Signaling Technology, catalog #4695), PAD4 (Abcam, catalog #ab96758, dilutetd 1:1000), and/or H3Cit (1:500; Abcam, catalog #ab5103) overnight at 4°C. Membranes were then washed and incubated with horseradish peroxidase-conjugated secondary antibodies (DAKO). Following washing in Tris-buffered saline containing 0.1% Tween-20 (TBST) for 10 min thrice,

the blots were visualized and imaged using an enhanced chemiluminescence Plus Kit (Millipore Corporation, Billerica, MA) and a ChemiDoc™ Imaging System (Bio-Rad). The proteins were normalized to β-actin (Sigma, catalog #A2228) and the respective total protein content. Densitometric analysis of the blots was performed using the ImageJ software (NIH ImageJ, USA).

Determination of plasma total cholesterol and lipid levels

Mice were fasted overnight. The next day, blood from each mouse was collected in heparinized tubes by retroorbital venous plexus puncture. The plasma was separated and analyzed for total cholesterol and triglyceride levels (DiaSys #113009911923, #157109911923) using enzymatic colorimetric assays as per the manufacturer's protocol.

ELISA Assay for sVCAM-1, TNF-α and IL-1β

Overnight fasted plasma samples were used for determination of sVCAM-1, TNF-α (tumor necrosis factor-α), and IL-1β (interleukin-1β) with commercially available mouse ELISA kits (R&D Systems, VCAM-1 kit catalog #MVC00, TNF-α kit catalog #MTA00B, IL-1β kit catalog #MLB00C) according to the manufacturer's instructions.

Reference

1. Doddapattar P, Dev R, Ghatge M, Patel RB, Jain M, Dhanesha N, Lentz SR, Chauhan AK. Myeloid Cell PKM2 Deletion Enhances Efferocytosis and Reduces Atherosclerosis. *Circ Res*. 2022:130(9):1289-1305. doi: 10.1161/CIRCRESAHA.121.320704

Tables

		$\alpha 9^{WT}$ $BM \rightarrow Ldlr^{-/-}$	$\alpha 9^{Mye\text{-}KO}$ $BM \rightarrow Ldlr^{-/-}$	*p* value
Body weight (g)	Female	20.8 ± 0.6	19.0 ± 0.7	0.064

Table S1. Body weight after 4 weeks of feeding a high-fat "Western" diet (n=10 female mice/group). The data are expressed as mean ± SEM. Statistical analysis: unpaired two-tailed *t*-test.

	$\alpha 9^{WT}$ $BM \rightarrow Ldlr^{-/-}$	$\alpha 9^{Mye\text{-}KO}$ $BM \rightarrow Ldlr^{-/-}$	*p* value
Total cholesterol (mg/dL)	855.1 ± 98.0	833.6 ± 98.2	0.870
Triglycerides (mg/dL)	167.6 ± 18.5	145.7 ± 12.0	0.236

Table S2. Total plasma cholesterol, plasma triglycerides concentrations were measured after 4 weeks of feeding a high-fat "Western" diet using enzymatic colorimetric assays according to the manufacturer's instructions. The data are expressed as mean ± SEM (n=6 mice/group). Statistical analysis: unpaired two-tailed *t*-test.

	$\alpha 9^{WT}$ $BM \rightarrow Ldlr^{-/-}$	$\alpha 9^{Mye\text{-}KO}$ $BM \rightarrow Ldlr^{-/-}$	*p* value
WBC (10^3 /µL)	13.5 ± 1.3	14.0 ± 1.8	0.836
RBC (10^6 /µL)	9.7 ± 0.1	9.2 ± 0.5	0.372
HGB (g/dL)	12.9 ± 0.2	12.4 ± 0.5	0.398
HCT (%)	49.2 ± 0.6	49.4 ± 1.8	0.944
PLT (10^3 /µL)	832 ± 25	841 ± 55	0.888
Neutrophils (10^3 /µL)	0.70 ± 0.18	0.80 ± 0.10	0.621

Table S3: Complete blood counts were obtained using the ADVIA 120 Hematology System. The data are expressed as mean ± SEM (n= 6 mice/group). Statistical analysis: unpaired two-tailed *t*-test.

Figures

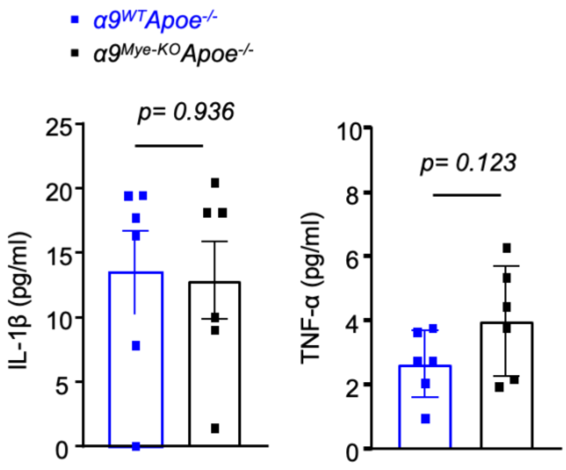

Figure S1. Quantification of plasma IL-1β and TNF-α cytokines levels. The data are expressed as mean ± SEM, n=6/group. Statistical analysis: unpaired two-tailed *t*-test.

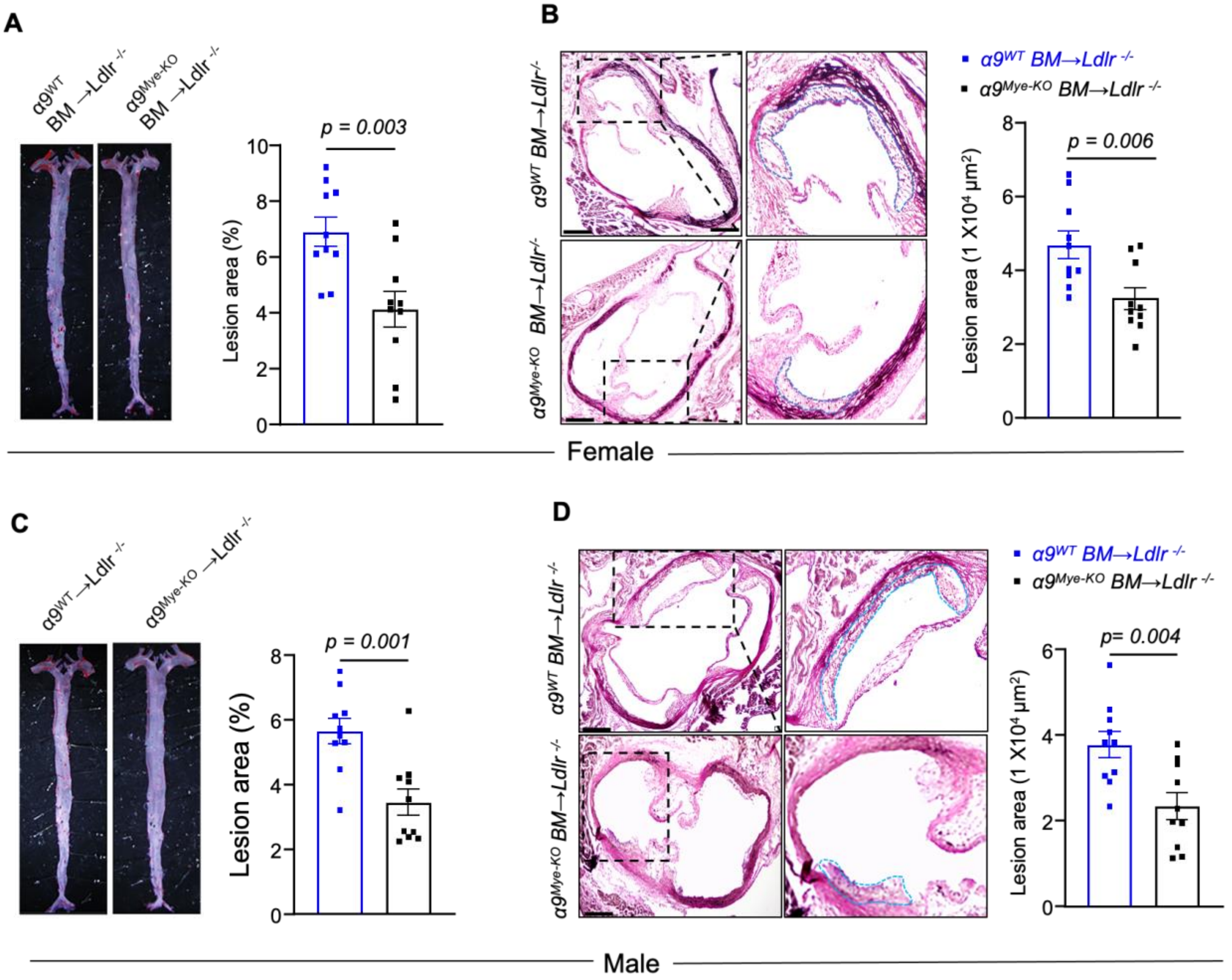

Figure S2. Genetic ablation of α9 in myeloid cells reduces early atherosclerosis in *Ldlr*$^{-/-}$ mice. Four weeks after BM transplantation, female and male mice were fed a high-fat diet for 4 weeks. **A&C**. Left shows representative photomicrographs and right shows quantification of *en-face* lesion areas in the whole aortae of female (n=10) or male (n=10) mice. **B&D**. Representative photomicrographs and quantification of the Verhoeff–Van Gieson staining of female (n=10) or male (n=10) mice. Scale bar, 500 μm. The data are presented as mean ± SEM. Statistical analysis: unpaired two-tailed *t*-test.

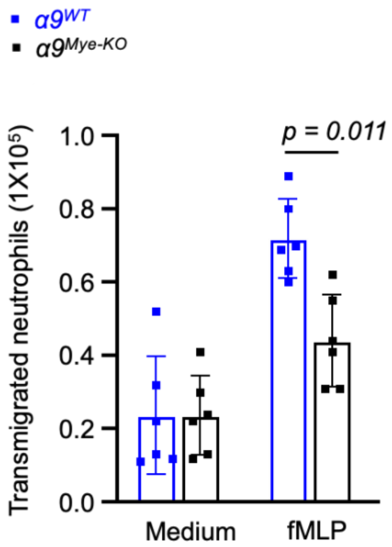

Figure S3. α9-deficient neutrophils migrate properly. BM-derived neutrophils were stimulated or not with fMLP for 1 h at 37°C and subjected to a transmigration assay (n=6/group). The data are from female mice and presented as mean ± SEM. Statistical analysis: unpaired two-tailed *t*-test.

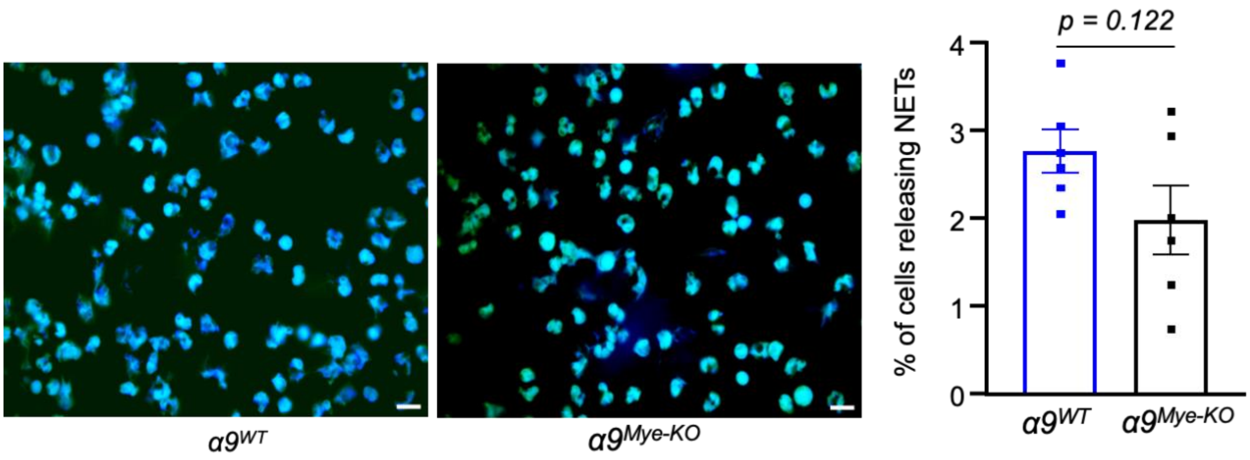

Figure S4. Neutrophils were incubated for 4 h at 37°C without VCAM-1 and stained with SYTOX Green nucleic acid strain (NETs-positive cells stained as green) and counterstained with Hoechst (blue). Scale bar, 10 μm. The percentage of neutrophils releasing NETs was calculated as the amount of NETs/number of neutrophils (Hoechst-positive) × 100. The data are expressed as mean ± SEM, n=6/group. Statistical analysis: unpaired two-tailed *t*-test.

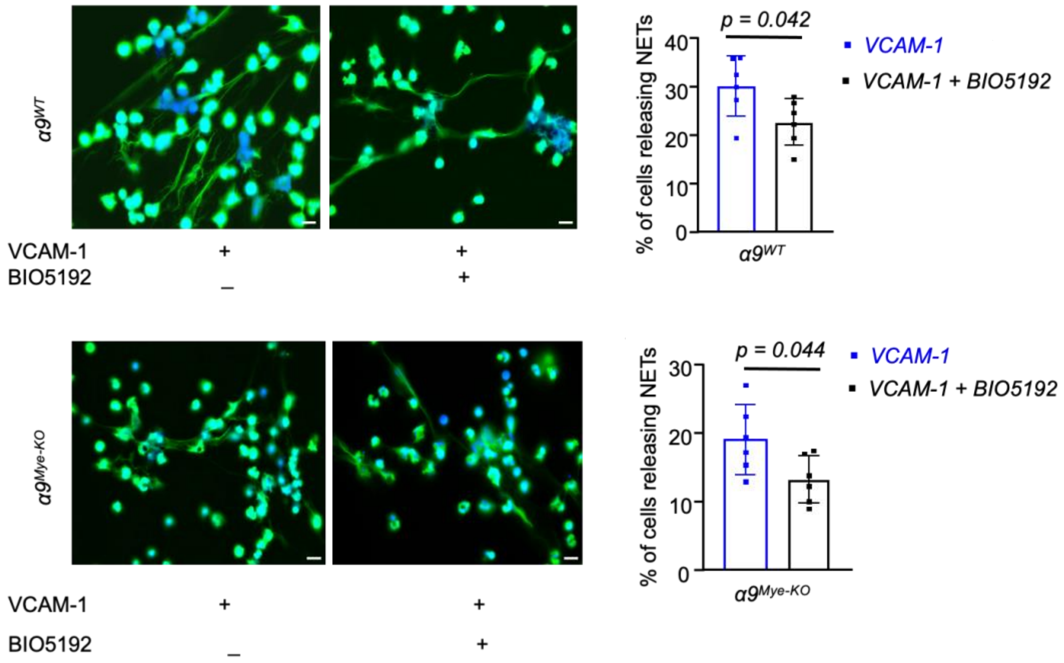

Figure S5. Inhibition of integrin α4β1 decreased NETs formation in both $\alpha 9^{WT}$ **and** $\alpha 9^{Mye\text{-}KO}$ **neutrophils**. Neutrophils were incubated at 37°C for 4 h with either VCAM-1 alone or with both VCAM-1 and BIO5192 (1 µM) and stained with SYTOX Green nucleic acid strain (NETs-positive cells stained as green) and counterstained with Hoechst (blue). Scale bar, 10 µm. The percentage of neutrophils releasing NETs was calculated as the amount of NETs/number of neutrophils (Hoechst-positive) × 100. The data are expressed as mean ± SEM, n=6/group. Statistical analysis: unpaired two-tailed *t*-test.
